# Inhibitory control and problem solving in early childhood: Exploring the burdens and benefits of high self‐control

**DOI:** 10.1002/icd.2297

**Published:** 2022-01-05

**Authors:** Alexandra Hendry, Mary A. Agyapong, Hana D'Souza, Matilda A. Frick, Ana Maria Portugal, Linn Andersson Konke, Hamish Cloke, Rachael Bedford, Tim J. Smith, Annette Karmiloff‐Smith, Emily J.H. Jones, Tony Charman, Karin C. Brocki

**Affiliations:** ^1^ Psychology Department Institute of Psychiatry, Psychology & Neuroscience, King's College London London UK; ^2^ Department of Experimental Psychology University of Oxford Oxford UK; ^3^ Department of Psychology & Newnham College University of Cambridge Cambridge UK; ^4^ Centre for Brain and Cognitive Development Birkbeck, University of London London UK; ^5^ Department of Psychology Uppsala University Uppsala Sweden; ^6^ Division of Neuropsychiatry, Department of Women's and Children's Health Center of Neurodevelopmental Disorders (KIND), Karolinska Institutet Stockholm Sweden; ^7^ Department of Child and Adolescent Psychiatry Institute of Psychiatry, Psychology & Neuroscience, King's College London London UK; ^8^ Department of Psychology University of Bath Bath UK

**Keywords:** divergent thinking, generativity, inhibitory control, problem‐solving, self‐regulation, toddlers

## Abstract

Low inhibitory control (IC) is sometimes associated with enhanced problem‐solving amongst adults, yet for young children high IC is primarily framed as inherently better than low IC. Here, we explore associations between IC and performance on a novel problem‐solving task, amongst 102 English 2‐ and 3‐year‐olds (Study 1) and 84 Swedish children, seen at 18‐months and 4‐years (Study 2). Generativity during problem‐solving was negatively associated with IC, as measured by prohibition‐compliance (Study 1, both ages, Study 2 longitudinally from 18‐months). High parent‐reported IC was associated with poorer overall problem‐solving success, and greater perseveration (Study 1, 3‐year‐olds only). Benefits of high parent‐reported IC on persistence could be accounted for by developmental level. No concurrent association was observed between problem‐solving performance and IC as measured with a Delay‐of‐Gratification task (Study 2, concurrent associations at 4‐years). We suggest that, for young children, high IC may confer burden on insight‐ and analytic‐aspects of problem‐solving.

## 1 INTRODUCTION

Inhibitory control (IC) is a multi‐faceted construct encompassing suppression of responses that conflict with goals, and resistance of temptation. High IC (versus low IC) is commonly framed as the more adaptive and cognitively‐mature response for young children (Diamond, 2013; Nigg, 2000) – in part due to the rapid developmental improvements in IC observed over the first 5 years of life (Hendry, Jones, & Charman, 2016; Petersen et al., 2016). For example, in a UK sample, 10‐month‐olds were able to resist touching a prohibited object for an average of only 3 s, but 16‐month‐olds were able to resist, on average, for 9 s (Hendry et al., 2021). By 24 months, more than half of a US‐sample were able to resist touching a prohibited object for the full 30 s before the prohibition was released. Similarly, amongst Dutch two‐ to three‐year‐olds, age was a significant predictor of latency to touch a prohibited snack or wrapped gift (Mulder et al., 2019), whilst Canadian 4‐year‐olds were more likely to wait for a larger reward than to choose an immediate, small reward compared with 3‐year‐olds (Prencipe & Zelazo, 2005). Furthermore, high IC amongst toddlers and pre‐schoolers is positively associated with a range of later outcomes including academic skills (Allan et al., 2014), physical health (Schlam et al., 2013), behavioural regulation (Buss et al., 2014; Eisenberg et al., 2004; Kochanska & Knaack, 2003), social competence and pro‐social behaviour (Kochanska, Murray, & Coy, 1997; Rhoades, Greenberg, & Domitrovich, 2009). However, these positive predictive associations may mask important nuances regarding contexts in which high IC is and is not adaptive in early childhood.

IC, as a component of self‐control and a key executive function, is related to emotional regulation (Bartholomew, Heller, & Miller, 2021; Carlson & Wang, 2007; Hudson & Jacques, 2014; Nigg, 2000). Amongst pre‐schoolers, individual differences in IC performance tend to correlate with individual differences on emotion regulation measures (Carlson & Wang, 2007; Hudson & Jacques, 2014). Imaging and genetic studies indicate that emotion regulation and inhibitory control involve common brain regions (Bartholomew et al., 2021; Liu et al., 2020) and are linked to similar genes (Liu et al., 2020). It has already been demonstrated that, even in early development, optimal emotional regulation does not entail high regulation across contexts (i.e., at trait level) but rather the ability to adapt to the situation (Eisenberg, Spinrad, & Eggum, 2010; Nigg, 2000). Here, we extend this reasoning to consider whether, for young children, IC confers advantage or disadvantage in other situations.

We focus our inquiry on a context where *low* IC has already been identified as sometimes advantageous for adults and older children: problem‐solving (Chrysikou, 2018). In adults, insight‐based problem‐solving performance – that is, requiring reframing a problem and generating creative solutions – can be enhanced by temporarily lowering IC, for example, through manipulating alertness (Wieth & Zacks, 2011), intoxication (Jarosz, Colflesh, & Wiley, 2012), or imposing prolonged IC demands (Cassotti et al., 2016; DeCaro & Van Stockum, 2018; Radel et al., 2015). In contrast, for analytic aspects of problem‐solving – such as evaluating solutions and systematically working through limited options, or when rapidly‐identified, seemingly intuitive solutions must be suppressed to allow for other solutions to be generated – low IC appears either unconnected to performance (Radel et al., 2015; Wieth & Zacks, 2011), or disadvantageous (Camarda et al., 2018; Cassotti et al., 2016; White & Shah, 2006). Extant research with children aged 4 years and older indicates that another aspect of executive function, cognitive flexibility, is inversely associated with divergent thinking (Vaisarova & Carlson, 2021) – but to our knowledge, the association between IC and problem‐solving in early childhood has not been examined.

The way we interpret variation in IC during early childhood matters: it matters for our theoretical models, for the messages given to parents and educators (e.g., framing low IC amongst children with developmental conditions as difference versus deficit), and for the priorities set for interventions. In developmental cognitive psychology research, the implicit context for assessment and evaluation of behaviour tends to prize efficient responses and high compliance (Hendry et al., 2016; White, 2013); such contexts may be orthogonal to insight and creativity. Efficient, compliant responses and analytic problem‐solving are also prized in schools (Bergold & Steinmayr, 2018; Poropat, 2009; Schonert‐Reichl & Hymel, 2007); this may account for some of the observed associations between academic outcomes and IC. To gain a more‐rounded understanding of the development of IC, and its role in day‐to‐day early childhood skills, requires an evaluation of what early individual differences in IC mean for both analytic and insight aspects of problem‐solving. As a starting point to this important line of enquiry, in two exploratory studies we examine associations between indices of IC and multiple aspects of performance on a novel problem‐solving task, amongst 18‐ to 48‐month‐olds.

IC can be measured through either observer report (commonly parent report in this age group), or performance measures. Parent report of IC in early childhood primarily pertains to a child's ability to control their behaviour when required, particularly in emotionally‐ or appetitively‐activating contexts (Gioia, Espy, & Isquith, 2002; Hendry & Holmboe, 2021; Putnam, Gartstein, & Rothbart, 2006; Rothbart et al., 2001). In terms of performance measures of IC suitable for very young children, broadly there are two types: directed global inhibition tasks, in which the participant is asked to hold off reaching for a desirable object and thus must inhibit a response in an emotionally‐ or appetitively‐activating context (such tasks include prohibition and delay of gratification tasks); and conflict or complex inhibition tasks in which participants are required to either inhibit a reaching response to a well‐primed (i.e., pre‐potent) location in favour of an alternative location, or to reverse a previously‐established stimulus–response mapping by changing from following one rule to following another (Hendry et al., 2021; Petersen et al., 2016). In this study, we focus on directed global inhibition tasks as our performance measure of IC, using tasks that are well‐established in the field (described further below), and which have been previously demonstrated to show longitudinal stability and/or associations with other measures of IC (Eigsti et al., 2006; Friedman et al., 2011; Hendry et al., 2021; Kochanska et al., 1996). These tasks are conceptually equivalent to the kinds of behaviours captured in parent‐report measures of IC – although associations between performance‐ and observer‐report measures of IC (and executive functions more broadly) are notoriously weak and may be sensitive to different, albeit complementary, aspects of IC (Toplak, West, & Stanovich, 2013). Further, directed global inhibition tasks have the advantage that the working memory demands of the tasks are minimal (as opposed to in conflict or complex inhibition tasks (Hendry et al., 2016; Petersen et al., 2016)) which avoids potential confound associations with problem‐solving performance.

## 2 STUDY 1

In this first exploratory study, we examine associations between indices of IC and multiple aspects of performance on a novel problem‐solving task, amongst 2‐ and 3‐year‐olds.

### 2.1 Methods

#### 2.1.1 Participants

Participants in this study were pooled from 3 large projects in the UK:

- The Studying Autism and ADHD Risks (STAARS) project: These participants were born full‐term and had no first‐degree family members with a diagnosis of Autism Spectrum Disorder (as confirmed through parent interview regarding family medical history). Twenty‐one of these participants were recruited as controls for the longitudinal STAARS study, and completed the Problem‐Solving Box task as part of a full‐day testing session comprising a battery of cognitive and behavioural assessments. The remainder were recruited as pilots and/or cross‐sectional controls and completed the Problem‐Solving Box task as part of a shorter (1–3 hr) visit.
- The London Down Syndrome (LonDownS) Consortium infant research stream: These participants completed the Problem‐Solving Box task as part of a full‐day testing session comprising a battery of cognitive and behavioural assessments.
- The Toddler Attentional Behaviours and Learning with Touchscreens (TABLET) project. Participants completed the Problem‐Solving Box task as part of the third longitudinal testing visit (2–3 hr session) comprising a battery of cognitive and behavioural assessments.

Participants were primarily recruited from the same volunteer database at the Birkbeck Centre for Brain and Cognitive Development, as well as social media, another volunteer database at Goldsmiths University of London InfantLab and personal contacts in the same region (London). Details of the highest level of maternal education was available for 73% of the sample (these data were not collected for the STAARS cross‐sectional participants, but we have no reason to expect the demographic profile of those participants to significantly differ from those from whom data were collected). These data are presented in Table 1. One additional participant originally contributed data but were not included due to administration error of the Problem‐Solving box task, and is not considered further. Developmental ability was assessed using the Mullen Scales of Early Learning (Mullen, 1995) for the UK sample and is reported for the Visual Reception scale (which is normed with a mean of 50 and standard deviation of 10) – see Table 1.

**TABLE 1:** Study 1 participant demographics and developmental level

	2‐year‐olds	3‐year‐olds
Mean age in months (SD)	24.83 (2.70)	40.66 (3.53)
*N* (boys)	49 (28)	53 (22)
Sample source	STAARS (*n* = 36), LonDownS (*n* = 13)	STAARS (*n* = 14), TABLET (*n* = 39)
Proportion with mothers educated to degree level or above^a^	83%	95%
Mean Mullen visual reception score (SD)	58.45 (11.33)	66.22 (9.97)

^**a**^ Remaining proportion educated to secondary level.

Ethical approval was granted for each contributing project as follows: The STAARS project, ethical approval from NHS London Research Ethics Committee (13/LO/0751) and King's College London PNM Research Ethics Subcomittee (HR‐16/17–3,509); the LonDownS Consortium infant research stream, ethical approval from NHS North West Wales Research Ethics Committee (13/WA/0194) and from the Birkbeck Psychological Sciences Ethics Committee (121,373, 13/1516); and the TABLET project, ethical approval from the Birkbeck Psychological Sciences Ethics Committee (171821). In all cases, parents provided informed consent and the study was performed in accordance with relevant ethical guidelines and regulations. None of the problem‐solving data presented below has been previously published.

#### 2.1.2 Performance measures of IC

Since there are no IC measures currently suitable for use across the entire infant‐to‐preschool period (Petersen et al., 2016), different IC tasks were used within each age band, to minimize ceiling and floor effects.

##### Glitter wand (two‐year‐olds)

Following the protocol described by Friedman et al. (2011), the participant was seated on a low table at a low table (STAARS) or on the parent's lap (LonDownS) with the experimenter seated at 90^○^. The experimenter drew the child's attention to an attractive toy (a Glitter Wand), made eye contact with the child, placed the toy on the table within reach of the child and said “Now [child's name], don't touch,” then moved away and pretended to be busy. The experimenter released the prohibition by saying “It's okay, you can touch it now” after the child touched the toy or after 30 s if the child did not touch. The dependent measure was the latency to touch the toy, with latencies ≥30 s indicating that the child did not touch the toy before the prohibition was released. Friedman et al. (2011) used this task to measure inhibitory control at ages 14 to 36 months, and found that IC profiles across the 14‐to‐36‐month period were predictive of scores on a “Common Executive Function” factor (derived, in part, from performance on a battery of IC tasks) at age 17 years (Friedman et al., 2011).

In our sample, the data showed a bimodal distribution so were reduced to two groups: high control (did not touch the wand before the prohibition was released) or low control (touched before the prohibition was released). The data were coded from video by four students blind to group status. In addition, an online rating (“touched/did not touch”) was provided by the researcher who administered the task. Agreement on whether the toddler touched or did not touch before the prohibition was reached was 100%. As this task was introduced after the two‐year visits had already begun in the STAARS study, data is missing for five toddlers. All other two‐year‐olds completed the task.

##### Snack delay (three‐year‐olds)

This task was adapted from Kochanska et al. (1996). The participant was seated at a low table with the experimenter seated at 180^○^. The experimenter placed a treat (a paw‐shaped dried fruit) under a transparent cup and instructed the participant to sit still with their hands on a placemat (with two handprints indicating placement) until the experimenter rang the bell to indicate that they could eat the treat. Four trials were administered, with delays of 10, 20, 30, and 240 s. Halfway through the delay, the experimenter lifted the bell but did not ring it. The experimenter restated the rule before each trial. Only one treat was placed under the cup for the first three trials, and three treats were placed on the fourth trial. During this last trial, the experimenter initiated a series of distractions (i.e., coughing, writing), and after she lifted the bell (but did not ring it) she left the room for a period of 90 s, and resumed the task when came back. The task took approximately 7 min to complete. Each trial was coded on a scale of 0 to 6 (0 = child ate the treat before the bell was lifted, 1 = ate the treat after the bell was lifted, 2 = touched the bell or cup before the bell was lifted, 3 = touched the bell or cup after the bell was lifted, 4 = removed both hands from the mat before the bell was lifted, 5 = removed both hands from the mat after the bell was lifted, and 6 = waited for the bell to ring). The sum of all trial scores was used as a continuous variable in the analysis below. Data were available for 39 three‐year‐olds as it was administered only in the TABLET study. Coding was done from video by an undergraduate student except for one case where the notes from the experimenter were used (because the video was not available). Ten videos were double coded by an independent coder. Inter‐rater reliability was 0.992 (95% CI: 0.969–0.998) assessed using a single measures 2‐way mixed model. Performance on the Snack Delay task has been previously found to correlate with performance on 4 other IC tasks at 26–41 months, and, as part of a composite score with those tasks, to show moderate longitudinal stability to age 43–56 months (Kochanska et al., 1996).

#### 2.1.3 Parent report of IC

To complement the behavioural measures, we also used parent report to gain insight into how IC skills are deployed in day‐to‐day life. Specifically, we used the IC scales of the Early Childhood Behaviour Questionnaire (ECBQ) (Putnam et al., 2006) with the two‐year‐old sample, and the Children's Behaviour Questionnaire (CBQ) (Rothbart et al., 2001) with the three‐year‐olds. As shown in SM2, the ECBQ‐IC scale pertains to a child's ability to control their behaviour when instructed, and can therefore be seen as conceptually equivalent to the Glitter Wand and Snack Delay tasks. As the behavioural repertoires of toddlers broaden with age, the CBQ‐IC scale also captures internally‐directed control (see SM2), but it has been previously demonstrated that ECBQ‐IC and CBQ‐IC scores show moderate homotypic continuity from toddlerhood (18–32 months) to early childhood (37 to 59 months) (Putnam, Rothbart, & Gartstein, 2008). In the CBQ and ECBQ parents are asked to report on how often they observed the child exhibiting certain behaviours during the last 2 weeks, on a scale of 1 (Never) to 7 (Always).

##### ECBQ‐IC (two‐year‐olds)

Forty parents completed either the six‐item IC scale from the ECBQ Short Form (Putnam et al., 2010) (STAARS) or the 12‐item ECBQ (Putnam et al., 2006) (LonDownS). Data were included only if a minimum of 70% of scale items were completed. ECBQ data were missing for six participants in the STAARS study, and three participants in the LonDownS study. Independent *t*‐tests indicated that there was no significant difference between participants with and without missing ECBQ‐IC data with regards to Success Score (*t*[47] = .542, *p* = .590), Generativity (*t*[46] = −1.277, *p* = .208), Persistence (*t*[46] = −.122, *p* = .904), or Perseveration (*t*[32] = 1.029, *p* = .188). Internal consistency of ECBQ‐IC data were good: Cronbach's alpha = .811 for the ECBQ short form and Cronbach's alpha = .883 for the full ECBQ.

##### CBQ‐IC (3‐year‐olds)

Forty‐two parents completed a minimum of five items from the six‐item IC scale of the CBQ Short Form (Putnam & Rothbart, 2006): CBQ data were missing for 1 participant in the STAARS study, not provided for a further 5 participants in the STAARS study due to not being in the protocol at the time of data collection, and missing for two parents in the TABLET study. Independent *t*‐tests indicated that there was no significant difference between participants with and without missing CBQ data with regards to Success Score (*t*[43] = −1.060, *p* = .295), Generativity (*t*[43] = 0.532, *p* = .598), Persistence (*t*[43] = 0.148, *p* = .883), or Perseveration (*t*[43] = 0.484, *p* = .631). Internal consistency of CBQ‐IC data were adequate: Cronbach's alpha = .720.

#### 2.1.4 Problem solving

**FIGURE 1:**
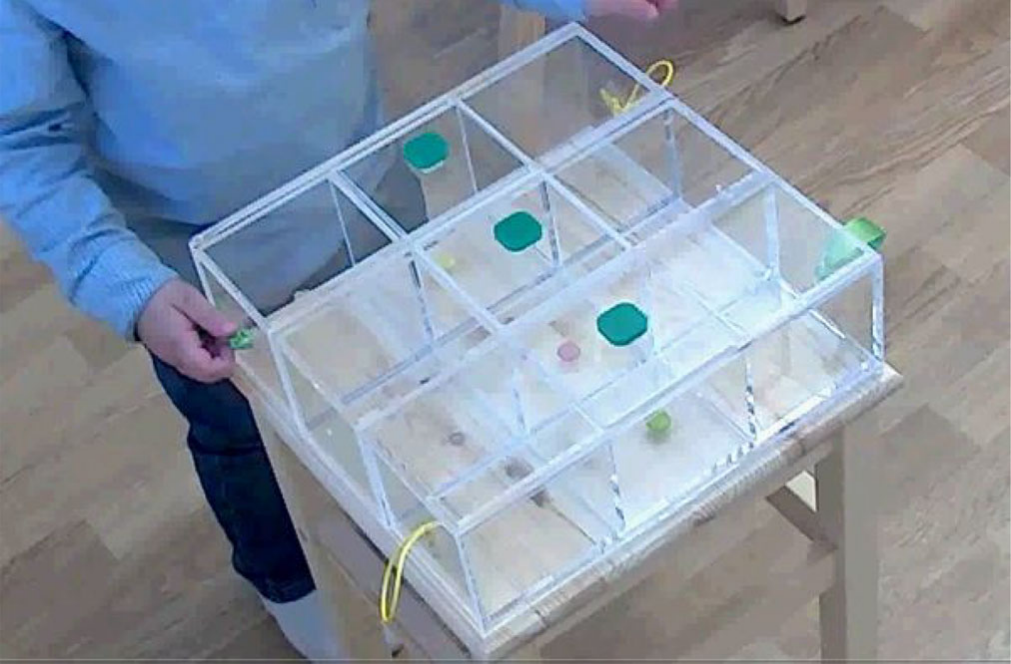
The Problem‐Solving Box

For all cohorts, our indices of problem‐solving were captured using a novel task, the **Problem‐Solving Box task**. This task was designed to present the kind of situation in which problem‐solving is used in day‐to‐day life for very young children (Burgess et al., 2006; Chaytor & Schmitter‐Edgecombe, 2003); the retrieval of desirable objects. The Problem‐Solving Box is a transparent acrylic box 30 cm × 30 cm × 10 cm with 3 compartments, each containing a small treat; e.g., *Smarties*, raisins or crackers – chosen either based on the parent's recommendation, or from a choice of 3 alternatives (Study 2). Built into the task are a set of physical constraints such that optimum outcome (retrieving all 3 rewards in 5 minutes) cannot be achieved purely through means‐end exploration: rather, previously‐successful or visually‐cued means‐end behaviours must be inhibited in favour of alternatives, and new strategies generated. Specifically, each compartment has a green knob attached, but only the central compartment lid can be lifted; the other two compartments are housed within sliding drawers each of which opens in the opposite direction from the other (see Figure 1 and https://osf.io/c65n9/ for further details). In this way, the Problem‐Solving Box task requires *insight‐based problem‐solving* (generating creative solutions). Note that participants are given credit for all solutions generated, not just for successful solutions. To be successful on the task, participants must persist in the face of set‐backs evaluate solutions and suppress rapidly‐identified solutions to allow for other solutions to be generated. In this way, the Problem‐Solving Box task also requires *analytic aspects of problem‐solving*).

##### Procedure

In the task warm up, the child was given one treat and allowed time to eat it. The Problem‐Solving Box was then brought into view of the child as the experimenter said “Here are more sweets for you. Can you get them?” The experimenter then moved to the side of the room. Once all three treats were retrieved or after 5 min, whichever was soonest, the experimenter terminated the task. If the child did not touch the box after 1 min, the experimenter gave a prompt to continue. Further prompts were administered after approximately every 30 s of non‐touching. If the child became distressed or tried to leave the room the task was terminated. This was the case for two participants. In both cases the task had been under way for more than 1.5 min and the participant had retrieved one reward. To avoid biasing the data to represent only children who can persist in a moderately‐challenging 5‐min task, these participants' data were included.

If any treats had not been retrieved at the end of the task time limit, the experimenter said “Well done, that was tricky wasn't it? This is how we open the box.” The experimenter then demonstrated each action. Any participants unable or unwilling to retrieve the rewards after demonstration would be excluded from analysis (on the basis that the task was either not motivating, or was beyond their motor control abilities); this did not occur for any participant.

##### Coding and data reduction

Coders rated toddlers' behaviour using the coding scheme in SM2. Across participants there was variation in overall task duration due to some participants quitting early (*n* = 2), retrieving all three treats within the time limit (*n* = 32), or technical or experimenter error (*n* = 3). To avoid penalizing these participants, Perseveration, Persistence, and Generativity scores were calculated from the first 3 min of the task for all participants. The overall Success Score was calculated from the full 5 min to maximize variation in the score, but the data were reviewed to ensure no child was given an artificially low score in the case of technical or experimenter error. The primary variables were:

- Generativity; number of distinct goal‐directed strategies attempted.
- Persistence; proportion of time spent on goal‐directed manipulation of the box.
- Perseveration; duration of time spent on the dominant strategy (specific to each participant) as a proportion of the time spent on goal‐directed manipulation. So that this variable was not distorted by non‐engagement with the task, children spending less than 10 s on goal‐directed manipulation during the first 3 min were excluded (*n* = 8).
- Success Score; derived from summing the latency to retrieve each reward and subtracting that from 900 (i.e., the amount of available time to collect each reward, summed) such that a score approaching 900 indicates successful problem‐solving, and a score of 0 indicates unsuccessful problem‐solving (see SM2 for further details).

Recordings were produced at a frame rate of 25 fps and coded with Mangold Interact v16. Data were coded by 1 lead coder (MA) and 3 additional student coders MH, BC, HC (see SM2 for inter‐rater reliability).

### 2.2 Analytic approach

We tested the associations between the continuous problem‐solving performance measures and our continuous measures of IC (Snack Delay, ECBQ‐IC and CBQ‐IC) using Pearson's correlations for the normally‐distributed variables (Generativity and Perseveration) and Spearman's Rho correlations for the non‐normally distributed variables (Success Score and Persistence). We tested the associations between problem‐solving performance and our bimodal measures of IC (Glitter Wand) using point‐biserial correlations, using Bootstrapping with 1,000 samples to accommodate non‐normal distributions. In addition, to check whether associations could be accounted for a common influence of developmental ability, we also report the results of partial correlations, controlling for developmental ability. Analyses were exploratory, thus in line with journal guidelines (Infant and Child Development, 2020) we do not include traditional inference criteria (i.e., *p* values), but rather interpret effect sizes greater than *r* = .2 – the minimum effect size of interest set for establishing the fail safe *N* by an influential meta‐analysis investigating relations between IC and academic outcomes (Allan et al., 2014). The data that support the findings of this study are available from the corresponding author upon reasonable request.

### 2.3 Results

Descriptive statistics for performance on the Problem‐Solving Box task and IC measures are shown in Table 2. As shown in Supplementary Table 1.2, Success Score is positively correlated with Generativity and Persistence, and negatively correlated with Perseveration. As shown in Supplementary Table 2.1, parent report of IC shows a weak positive association with concurrent performance scores at age 2 years, but the association between parent report and performance measures of IC at age 3 years is very weak (i.e., not interpretable). As shown in Supplementary Table 3.1, Success Score, Generativity and Persistence (but not Perseveration) show a significant positive association with age.

**TABLE 2:** Descriptive statistics of key variables derived from performance on the Problem‐Solving Box task and IC measures for Study 1 participants

		2‐year‐olds	3‐year‐olds
	*N*	49	53
Success score	Mean	224.63	378.47
	SD	235.03	243.48
	Range	0–787	0–819
	% at floor	31%	9%
	% at ceiling^a^	0%	0%
Generativity	Mean	5.35	8.96
	SD	3.44	2.70
	Range	0–13	3–15
	% at floor^b^	15%	0%
	% at ceiling^c^	0%	2%
Persistence	Mean	.18	.39
	SD	.15	.17
	Range	.00–.49	.05–.77
	% at floor	15%	0%
	% at ceiling^d^	0%	0%
Perseveration	Mean	.43	.49
	SD	.19	.19
	Range	.19–1.00	.16–.93
	% at floor^e^	3%	0%
	% at ceiling^f^	0%	0%
Proportion in low Control group^g^		45%	56%
Proportion in high Control group^g^		55%	44%
Mean (SD) IC score (ECBQ/CBQ)		4.71 (.92)	4.68 (.95)

^a^ Ceiling for Success Score set at 870; each reward retrieved in under 10 s.

^b^ Floor set at 0.

^c^ The observed maximum level of Generativity (15 unique strategies attempted) was set as the ceiling level, to provide a more conservative estimate than using the total number of unique strategies observed across participants (23).

^d^ Ceiling set at .9 (engaged in goal‐directed behaviour for at least 90% of the time), to provide a more conservative estimate than using the total possible level of Persistence (1.0).

^e^ As Perseveration is negatively associated with performance, floor is set at 1.0 (100% of goal‐directed behaviour spent on a single strategy).

^f^ Ceiling set at the absolute observed minimum (.02); 2% of manipulation time engaged in a single behaviour.

^g^ Based on Glitter Wand performance in the 2‐year‐old sample, and Snack Delay performance in the 3‐year‐old sample. Snack Delay data shown as dichotomous scores based on median split for comparison purposes. Analyses below use continuous Snack Delay data: Mean Snack Delay scores = 21.69, SD = 2.25.

#### 2.3.1 Associations between problem‐solving and IC

**FIGURE 2:**
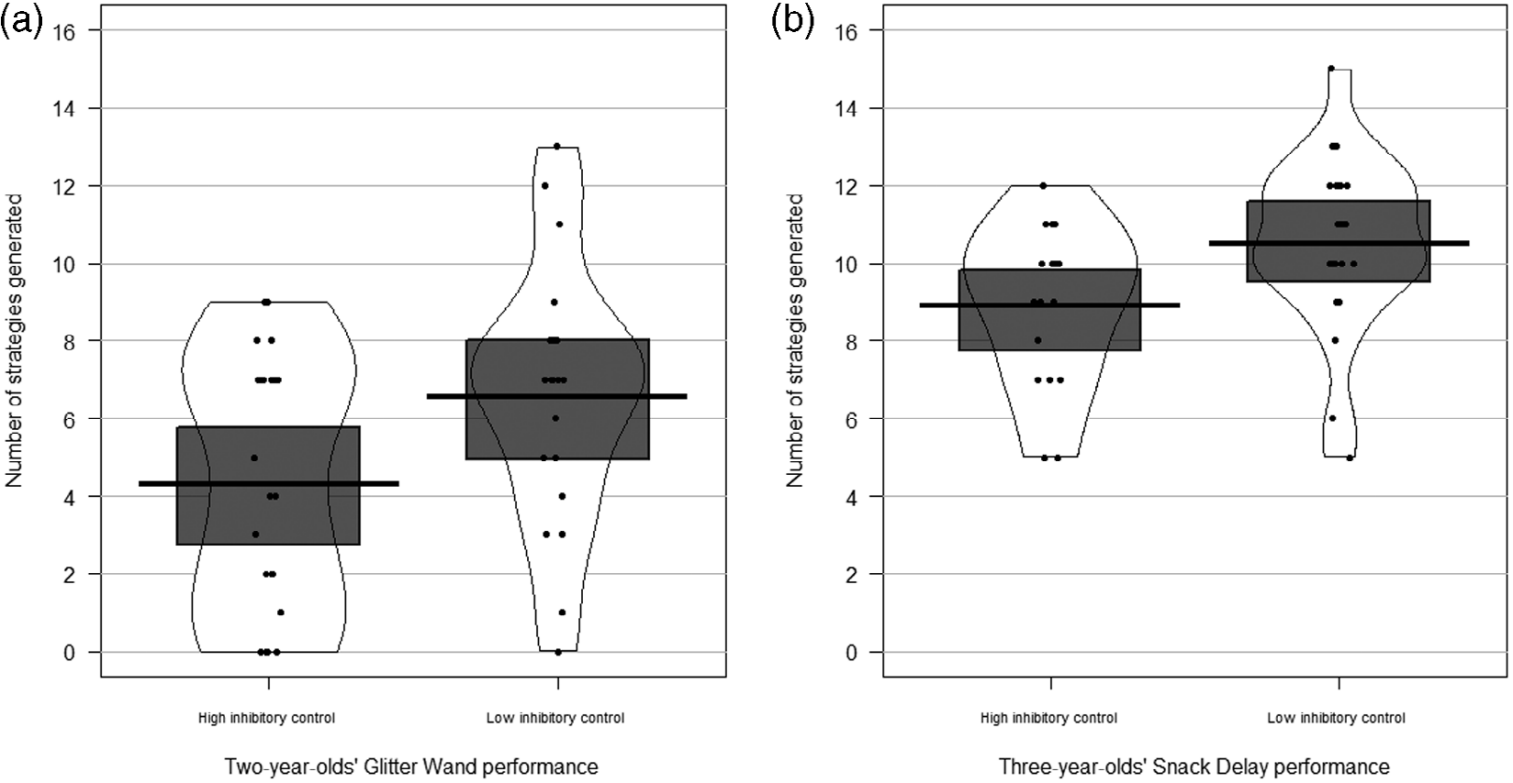
Generativity on the Problem‐Solving Box task by: (a) 2‐year‐olds' Glitter Wand performance; and (b) 3‐year‐olds' Snack Delay performance (median split for illustrative purposes only). The thick black line indicates the group mean, the dark grey box the 95% Confidence Interval and the outer line the bean density

As shown in Table 3 and Figure 2, at both 2 and 3 years, IC on prohibition‐type performance measures shows a moderate negative concurrent association with Generativity. In addition, performance IC shows a weak negative concurrent association with overall problem‐solving performance at age 2 years, but the confidence interval includes 0. Results are broadly unaffected by controlling for developmental ability.

**TABLE 3:** Associations between Problem‐Solving performance and IC

	IC measure	Age (n)	Success score	Generativity	Persistence	Perseveration
Performance measures of IC	Glitter wand	2‐year‐olds (44)	−.25 (−.29) [−.52, .06]	−.33 (.34) [−.55, −.03]	−.17 (.18) [−.43, .15]	.19 (.19) [−.19, .53]
	Snack delay	3‐year‐olds (39)	−.14 (−.16) [−.43, .19]	−.42 (−.43) [−.63, −.14]	.04 (.03) [−.33, .39]	.10 (.10) [−.21, .36]
Parent‐report measures of IC	ECBQ‐IC	2‐year‐olds (40)	−.01 (−.07) [−.34, .31]	−.13 (−.13) [−.37, .13]	.17 (.23) [−.17, .44]	−.13 (−.15) [−.45, .19]
	CBQ‐IC	3‐year‐olds (42)	−.40 (−.43) [−.65, −.10]	−.25 (−.22) [−.49, .09]	.31 (.16) [.05, .56]	.35 (.31) [.10, .55]

*Note*: Cell values show uncorrected correlation co‐efficient with 95% confidence interval in square parentheses, and correlation co‐efficient controlling for Mullen visual reception normed score in round parentheses.

Abbreviations: CBQ, children's behaviour questionnaire; ECBQ, early childhood behaviour questionnaire; IC, inhibitory control.

**FIGURE 3:**
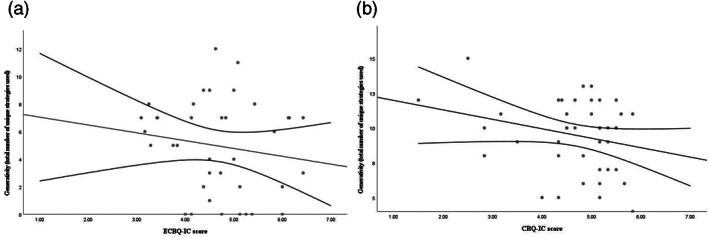
Generativity on the Problem‐Solving Box task by parent‐reported IC scores at: (a) age 2 years; and (b) age 3 years

As shown in Table 3 and Figure 3, for the two‐year‐olds no interpretable concurrent associations between parent report of IC and problem‐solving performance are observed. For the three‐year‐olds, IC shows a moderate negative association with overall problem‐solving performance. There is also a weak negative association with Generativity, but the confidence interval includes 0. Unlike for the performance measures, there is a weak positive concurrent association between parent‐reported IC and both persistence (although this becomes uninterpretable after controlling for developmental ability) and perseveration at age 3 years.

### 2.4 Discussion

In this exploratory study of 102 English 2‐ and 3‐year‐olds, we found evidence that IC is concurrently negatively associated with generativity specifically, and problem‐solving success more generally. Generativity‐IC associations were most convincing for performance measures of IC (at both ages 2 and 3 years), whereas problem‐solving success‐IC associations were most convincing for parent‐report of IC (for 3‐year‐olds only). Perseverative behaviour (i.e., getting “stuck” on one mode of response) was also associated with higher parent‐reported IC at age 3 years (only). We reflect further on these results in the overall discussion section below.

## 3 STUDY 2

### 3.1 Methods

#### 3.1.1 Participants

Participants in this study were part of a longitudinal study in Sweden starting at 10 months of age, with assessment of different aspects of caregiving behaviours and self‐regulatory functions at 10, 18, 36, 48, and 72 months. The Problem‐Solving Box task was part of the 48 months assessment. Inhibitory control performance was collected at 18 and 48 months (from the same participants, longitudinally). Participants were recruited through the birth registry of Uppsala. Demographic data (highest level of maternal education) was available for 95% of the sample. Developmental ability was assessed using the Wechsler Preschool and Primary Scale of Intelligence – Third Edition (WPPSI ‐ III) (Wechsler, 2002) Block scale (which is normed with a mean of 10 and standard deviation of 3). These data are presented in Table 4.

**TABLE 4:** Study 2 participant demographics

	18‐month visit	4‐year visit
Mean age in months (SD)	17.72 (.52)	48.08 (.61)
*N* (boys)	81 (44)	84 (45)
Proportion with mothers educated to degree level or above^a^	90%	90%
Mean Wechsler block scale score (SD)	11.81 (3.27)	11.79 (3.28)

^**a**^ Remaining proportion educated to secondary level.

Eight additional participants originally contributed problem‐solving data. Three were excluded due to administration error, one due to diagnosis of a genetic condition, and four due to the child not attempting the problem‐solving task or quitting early to use the toilet. For the four participants excluded due to not attempting/completing the problem‐solving box, the mean latency to touch on the IC task at 18 months (8.50 s) was within 1 standard deviation of the mean latency to touch for all included participants (10.53 s, SD = 12.08). All four excluded participants were given a score of 1 (high IC) on the delay of gratification task at 48 months, versus 72% of included participants. Thus, there is no clear evidence to suggest that the IC skills of participants who did not attempt or complete the problem‐solving box significantly differ from included participants, and these participants are not considered further.

Ethical approval was granted by the local ethics committee in Uppsala Sweden (EPN; Dnr. 2013/241). In all cases, parents provided informed consent and the study was performed in accordance with relevant ethical guidelines and regulations. The data on four‐year‐olds' IC performance has been previously presented in a study examining associations between maternal sensitivity and later self‐regulation (Frick, Forslund, & Brocki, 2019), and 18‐month‐olds' IC performance in a study examining the development of self‐regulation (Frick et al., 2018). None of the problem‐solving data presented below has been previously published.

#### 3.1.2 Performance measures of IC

##### Glitter wand (18‐month visit)

The protocol and coding approach outlined in Study 1 was followed, with the exception that infants were seated in a high chair placed at a normal table. Three participants did not contribute Glitter Wand data at 18 months: two did not attend the 18‐month‐visit and one infant was excluded due to parental interference.

##### Delay of gratification (four‐year visit)

This task was adapted from Mischel, Shoda and Rodriguez (Mischel, Shoda, & Rodriguez, 1989) using the protocol described in (Frick et al., 2019). In brief, participants were told that if they could wait until the researcher returned, they could have 10 treats, but that if they did not want to wait they could ring a bell and receive two treats immediately. The researcher then left the room, returning if the bell was rung, or after 3 min if the bell was not rung. Children who rang the bell within the three‐minute period, or began eating the snacks, were given a score of 0 (low IC); children who were able to wait for the full 3 min were given a score of 1 (high IC). Data were coded by a trained researcher, who noted no ambiguous behaviour. Data were available for 83 four‐year‐olds: one infant was excluded as they were not willing to participate in the task. Performance on the Delay of Gratification task has previously been found to predict a range of IC‐related developmental outcomes as well as performance on cognitive measures of IC in adolescence and adulthood (Eigsti et al., 2006).

#### 3.1.3 Problem solving

The Problem‐Solving Box task was administered and coded following the protocol outlined in Study 1. Data were coded by 1 lead coder (MA) and 1 additional student coder (NK) (see SM2 for inter‐rater reliability). Forty‐six participants retrieved all three treats within the time limit.

### 3.2 Analytic approach

**FIGURE 4:**
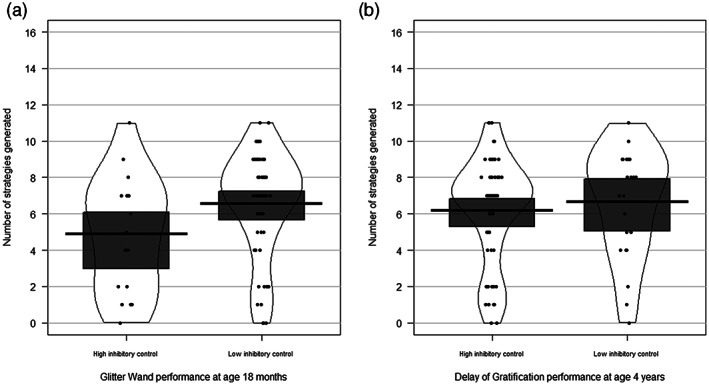
Generativity on the Problem‐Solving Box task at age 4 years by: (a) Glitter Wand performance at age 18 months; and (b) Delay of Gratification performance at age 4 years. The thick black line indicates the group mean, the dark grey box the 95% Confidence Interval and the outer line the bean density

As per Study 1, associations between problem‐solving performance and our bimodal measures of IC (Glitter Wand, Delay of Gratification) were calculated using point‐biserial correlations, using Bootstrapping with 1,000 samples to accommodate non‐normal distributions (Success Score, Persistence, and Perseveration variables). In addition, to check whether associations could be accounted for a common influence of developmental ability, we also report the results of partial correlations, controlling for developmental ability (Figure 4).

### 3.3 Results

Descriptive statistics for performance on the Problem‐Solving Box task and IC measures are shown in Table 5.

**TABLE 5:** Descriptive statistics of key variables derived from performance on the Problem‐Solving Box task and IC measures for Study 2 participants

		4‐year‐olds
	*N*	84
Success score	Mean	413.50
	SD	289.93
	Range	0–835
	% at floor	16%
	% at ceiling^a^	0%
Generativity	Mean	6.24
	SD	3.00
	Range	0–11
	% at floor^b^	5%
	% at ceiling^c^	0%
Persistence	Mean	.25
	SD	.13
	Range	.00–.52
	% at floor	5%
	% at ceiling^d^	0%
Perseveration	Mean	.13
	SD	.08
	Range	.02–.47
	% at floor^e^	0%
	% at ceiling^f^	4%
Proportion in low control group^g^		28%
Proportion in high control group^g^		73%

^a^ Ceiling for Success Score set at 870; each reward retrieved in under 10 s.

^b^ Floor set at 0.

^c^ The observed maximum level of Generativity (15 unique strategies attempted) was set as the ceiling level, to provide a more conservative estimate than using the total number of unique strategies observed across participants (23).

^d^ Ceiling set at .9 (engaged in goal‐directed behaviour for at least 90% of the time), to provide a more conservative estimate than using the total possible level of Persistence (1.0).

^e^ As Perseveration is negatively associated with performance, floor is set at 1.0 (100% of goal‐directed behaviour spent on a single strategy).

^f^ Ceiling set at the absolute observed minimum (.02); 2% of manipulation time engaged in a single behaviour.

^g^ Based on Delay of Gratification performance.

#### 3.3.1 Associations between problem‐solving and IC

As shown in Table 6 and Figure 4, there is no interpretable association between problem‐solving performance and concurrent IC at age 4 years. However, there is a weak negative association between high IC at 18 months and generativity at age 4 years, even after controlling for developmental ability.

**TABLE 6:** Associations between Problem‐Solving performance and IC

IC measure	Age (n)	Success score	Generativity	Persistence	Perseveration
Glitter wand	18‐month‐olds^a^ (81)	−.12 (−.13) [−.32, .11]	−.23 (−.26) [−.47, −.01]	−.16 (−.18) [−.40, .09]	−.06 (−.06) [−.29, .19]
Delay of gratification	4‐year‐olds (83)	−.10 (−.11) [.29, .12]	−.07 (−.07) [−.28, .16]	−.17 (.−19) [−.37, .05]	−.14 (−.13) [−.35, .09]

*Note*: Cell values show uncorrected correlation co‐efficient with 95% confidence interval in square parentheses, and correlation co‐efficient controlling for Weschler block normed score in round parentheses.

^a^ Longitudinal association to Problem‐Solving Box performance at age 4 years.

### 3.4 Discussion

In this exploratory study of 84 Swedish children, seen longitudinally at 18 months and 4 years old, we did not find evidence of a concurrent association between IC and problem‐solving performance age 4. However, strong IC at 18 months was predictive of less generativity on a problem‐solving task at age 4 years. Below, we reflect on how these results converge and diverge from those of Study 1, and the implications of our findings for research and practice.

## 4 OVERALL DISCUSSION

Two exploratory studies investigated whether IC skills, as indexed by performance on prohibition and delay of gratification‐type directed global inhibition measures, and related parent‐report of trait IC, confers advantage and/or disadvantage with regards to problem‐solving amongst children age 4 years and under. Our results indicate that high IC in early childhood may be disadvantageous for both insight‐based and analytic problem‐solving. Our metric of insight‐based problem‐solving was the number of distinct strategies generated. Our metrics of analytic problem‐solving were overall problem‐solving success (being able to retrieve all 3 rewards within a limited time period), persistence (proportion of time spent in goal‐directed behaviour) and (inversely) perseveration (proportion of time spent on the dominant strategy). In both studies, a negative association was observed between generativity and IC as measured by the ability to comply with a prohibition to touch an appealing toy, or reach for an appealing treat. This association was observed for concurrent associations with problem‐solving performance at age 2 and 3 years (Study 1, UK sample), and longitudinal performance from 18‐month IC performance to 4‐year problem‐solving performance (Study 2, Sweden sample). In addition, in the UK 3‐year‐old sample only, high parent‐reported IC was associated with poorer overall problem‐solving success, and more perseverative behaviour.

In Study 2, our IC measure was not concurrently associated with any metric of problem‐solving at age 4 years. Given the predictive associations between IC at 18 months and generativity at 4 years found within the same sample, one possibility is that the negative impact of IC on generativity is weakened by the age of 4 years. However, there is little in the literature to foreshadow this account of a developmental shift in IC‐generativity associations, given that negative IC‐generativity associations have previously been observed in adulthood (DeCaro & Van Stockum, 2018; Jarosz et al., 2012; Radel et al., 2015; Wieth & Zacks, 2011). Research with adults does indicate that null and even positive IC‐generativity associations are observed when the nature of the problem‐solving is analytical or requires suppression of rapidly‐identified, seemingly intuitive solutions (Camarda et al., 2018; Cassotti et al., 2016; Radel et al., 2015; White & Shah, 2006; Wieth & Zacks, 2011); but if there had been a developmental shift in the way in which 4‐year‐olds were approaching the problem‐solving task (i.e., using an analytic rather than insight‐based approach) this would not explain why negative longitudinal associations were observed from IC at 18 months to generativity at age 4 years.

Another possibility is that, in Study 2, negative associations with generativity at age 4 years were found for IC measured at 18 months but not at 4 years due to differences in the IC measures at the two time‐points. The 18‐month measure was a prohibition task (just as in both time‐points in Study 1, where negative IC‐generativity associations were also observed) whereas the 4‐year IC measure was a Delay of Gratification task in which participants were asked to decide for themselves whether to wait to receive the preferred, larger reward or to immediately select the smaller reward. Previous work has indicated that 4‐year‐olds who choose the delayed reward in this task do so by integrating a third‐person and first‐person perspective, as well as by engaging IC skills (Prencipe & Zelazo, 2005). Potentially, the advantage conferred to generativity by the ability to shift or integrate perspectives balanced out some of the disadvantage conferred by having high IC.

There was limited evidence of high IC conferring benefits to problem‐solving. In Study 1, high parent‐reported IC was weakly associated with increased persistence – but the association became very weak after accounting for developmental ability. Parent‐reported IC– persistence associations were found for the CBQ (used with 3‐year‐olds) but not the ECBQ (used with 2‐year‐olds). ECBQ‐IC items focus on compliance to prohibition, whereas CBQ‐IC includes more internally‐directed control. ECBQ‐IC scores show a weak positive association with concurrent performance scores, but CBQ‐IC scores do not. Thus, we conjecture that parent‐report and performance measures focusing on compliance are not sensitive to those aspects of IC that associate with persistence, and which may also be involved in general developmental ability. Note that parent‐reported IC at 3 years was also associated with greater perseveration – that is, getting “stuck” on a particular mode of responding. Thus, even though internally‐directed IC may confer benefits in terms of general persistence, the practical advantages of these benefits may be limited by high‐IC children being more likely to persevere with ineffective behaviours.

### 4.1 Strengths and limitations

A strength of our study was the inclusion of the Problem‐Solving Box task, a novel open‐ended task with low social and language demands. This task enables participants to demonstrate their spontaneous problem‐solving style, by accommodating a variety of possible behavioural responses (Chen & Siegler, 2000), and provides an opportunity to dissociate areas of relative strength and difficulty within one 5‐min task.

Another strength was our ability to (partially) conceptually replicate findings across multiple, reasonably‐powered samples. Nevertheless, as this was an exploratory study, further replication is required before firm conclusions can be drawn. Further, our participants were primarily from highly‐educated backgrounds, with no known genetic or developmental condition, and results may not generalize to other populations. Priorities for future research include examining whether advantages in problem‐solving are observed for toddlers in populations linked to low IC, such as those with, or on a pathway towards, ADHD (Pauli‐Pott & Becker, 2011; White & Shah, 2006).

A limitation of the study is that, due to the lack of IC tasks suitable across the age‐range of interest (Petersen et al., 2016) IC measures varied with age. This constrains conclusions about how IC–problem‐solving associations change across early childhood. In particular, our interpretation of the null association between generativity and Delay of Gratification performance at 4 years is largely conjectural. Relatedly, this study focused on IC as measured by performance on directed global inhibition tasks and conceptually‐related parent‐report measures. Future research might consider whether performance on conflict or complex‐inhibition tasks also confer burden and benefit, as well as how IC interacts with related executive functions such as working memory to influence performance on day‐to‐day tasks.

### 4.2 Implications for theory

Our data support the argument that the optimal level of IC is context‐dependent (Chrysikou, Weber, & Thompson‐Schill, 2014), and that high IC is not inherently better than low IC in early childhood. Researchers have argued that the protracted developmental time‐course of the prefrontal cortex and the associated slow development of IC must have some evolutionary advantages (Gopnik, 1803; Thompson‐Schill, Ramscar, & Chrysikou, 2009). One possibility is that the limitations of childhood prefrontal cortex may prevent IC from being over‐deployed until we have had time (i.e., in our early 20s) to learn when it is appropriate to inhibit (i.e., in high‐risk or highly‐constrained contexts) and when not (i.e., when problem‐solving or exploiting all possibilities in a safe learning context) (Gopnik, 1803). In addition, because low IC is more conducive to an unregulated maximization approach (in which the most frequently‐detected pattern or rule is applied), rather than to the more cognitively‐sophisticated strategic probability matching approach (using top‐down control to flexibly deploy a rule based on the likelihood of it applying to the specific event), low IC may facilitate learning when input is noisy or incomplete (as with much language input) or conflicts with prior experience (as with some abstract principles) (Gopnik, Griffiths, & Lucas, 2015; Ramscar & Gitcho, 2007; Thompson‐Schill et al., 2009). Noisy and incomplete stimuli or task demands, and conflicts with prior experience, are common in many day‐to‐day problems – and the Problem‐Solving Box task was designed specifically to mimic this. This study therefore provides a first hint that the cognitive style associated with low IC may facilitate problem‐solving in early childhood.

### 4.3 Implications for practice

Our findings highlight the importance of considering the context‐specific advantages and disadvantages of individual differences in IC. Whilst further research is certainly needed, science communicators and the research and clinical communities more generally should in the meantime be wary of framing low IC in early childhood as a problem to be either outgrown or intervened upon, and instead discuss both the opportunities and limitations that low or high IC may afford for each child as an individual. For example, does a toddler who has difficulties waiting find creative tasks engaging? Does a highly‐compliant toddler show difficulty with considering new ways of tackling a problem? These questions are important in considering in which contexts a child is likely to thrive, and where they may benefit from extra support so that, over time, they develop strong self‐esteem and the ability to flexibly up‐ or down‐regulate their inhibitory response to match current task demands. Extending this idea further, we argue that research is needed into the potential negative impacts of interventions which seek to promote IC in early childhood (whether under the guise of compliance and “good” behaviour, or performance on cognitive tasks). If, as we suggest above, low IC may be an evolutionarily–adaptive strategy for some types of learning, are there hidden costs associated with accelerating IC development in young children, either through explicit intervention or through creating environments which have high IC demands (such as if a preschool setting were to emphasize waiting, rule‐following and rote learning over discovery learning and free play)? To evaluate this question effectively, both intervention‐evaluations, and observational studies should avoid using only outcome measures where high scores are contingent on compliance and analytic‐problem solving (which is likely to inflate positive IC‐outcome associations) and also use outcomes that are sensitive to insight and creativity.

## AUTHOR CONTRIBUTIONS

**Alexandra Hendry:** Conceptualization; data curation; formal analysis; investigation; methodology; visualization; writing – original draft; writing – review and editing. **Mary A Agyapong:** Investigation; writing – review and editing. **Hana D'Souza:** Investigation; supervision; writing – review and editing. **Matilda Frick:** Investigation; supervision; writing – review and editing. **Ana Maria Portugal:** Investigation; writing – review and editing. **Linn Andersson Konke:** Investigation; writing – review and editing. **Hamish Cloke:** Investigation; writing – review and editing. **Rachael Bedford:** Investigation; supervision; writing – review and editing. **Tim Smith:** Investigation; supervision; writing – review and editing. **Annette Karmiloff‐Smith:** Funding acquisition. **Emily Jones:** Conceptualization; data curation; investigation; project administration; supervision; writing – review and editing. **Tony Charman:** Conceptualization; funding acquisition; supervision; writing – review and editing. **Karin Brocki:** Funding acquisition; project administration; supervision; writing – review and editing.

## Supporting information


**Data S1.** Supporting information.

## Data Availability

The data that support the findings of this study are available from the corresponding author upon reasonable request.
