# Supplementary material for: Inhibitory control and problem solving in early childhood: Exploring the burdens and benefits of high self‐control
Source: Infant Child Dev. 2022 Jan 5;31(3):e2297. doi: 10.1002/icd.2297 (PMC9364682; doi:10.1002/icd.2297)
Supplement: Supplementary file 1 — Data S1. Supporting information. [file ICD-31-0-s001.docx]

# Supplementary Materials 1: Problem-Solving Box task details

Videos were coded according to the coding scheme indicated in below where each code indicates a continuous, mutually-exclusive behaviour. Behaviours included in the strategy count are indicated in italics. Behaviours considered as non-goal directed sensory-motor behaviours are indicated in bold. Note that, as detailed in Supplementary Table S1, a given strategy (e.g. lid lifting) was considered distinct in each location to which it was applied (e.g. left-hand, central and right-hand lids).

Supplementary Table 1.1 Behavioural codes for the Problem-Solving Box Task

| Code | Notes |
| --- | --- |
| *push down left lid* | Child presses down on either the green knob or the compartment itself |
| *push down centre* | Child presses down on either the green knob or the compartment itself |
| *push down right* | Child presses down on either the green knob or the compartment itself |
| *peeling up (anywhere)* | Child uses nails/fingertips to exert pressure on a small part of the box, generally near to a join in materials, seemingly to peel off a layer |
| *squeezing/pinching (anywhere)* | Child uses fingertips and/or thumbs to exert pressure on a small part of the box, seemingly to interrogate its physical properties |
| *lift lid – left* | Child pulls on the green knob on the left of the box. This action will not be successful so is coded any time the child applies some force (gripping/pulling) |
| *lift lid – centre* | Child pulls on the green knob in the centre of the box (the lid will lift when pulled) |
| *lift lid – right* | Child pulls on the green knob on the right of the box. This action will not be successful so is coded any time the child applies some force (gripping/pulling) |
| *pull string – left* | Child touches and exerts force on the string attached to the drawer on the left side of the box. This action will not be successful so is coded any time the child applies some force. Just fiddling with the string is coded as touching box but no obvious manipulation. |
| *pull string – right* | Child touches and exerts force on the string attached to the drawer on the right side of the box. This action will not be successful so is coded any time the child applies some force. Just fiddling with the string is coded as touching box but no obvious manipulation. |
| *pull ribbon – left* | Child touches and exerts force on the ribbon. If child exerts force but doesn’t manage to move the drawer (i.e. because pulling from an angle) still code as a pull. |
| *pull ribbon – right* | Child touches and exerts force on the ribbon. If child exerts force but doesn’t manage to move the drawer (i.e. because pulling from an angle) still code as a pull. |
| *push drawer – left* | Child pushes on drawer (rather than pulling string/ribbon) |
| *push drawer – centre* | Child pushes on drawer (rather than pulling string/ribbon) |
| *push drawer – right* | Child pushes on drawer (rather than pulling string/ribbon) |
| *push whole unit* | Child uses arms, legs or whole body to move whole unit away from their body |
| *lift whole unit* | Child uses arms to lift unit (may achieve only a small lift) |
| *pull whole unit* | Child uses arms, legs or whole body to move whole unit away towards their body |
| *pressing down on unit* | Child uses hands, arms or body to push down on the unit (if the pressing down directly precedes a climbing action, code as part of climbing) |
| *trying to access through central compartment* | Child places fingers/hands against the wall of the central compartment adjacent to the other compartments. Note that this behaviour looks different to reaching back into the central compartment as they are exploring the boundaries of the compartment |
| *peeling up with tool (anywhere)* | Child uses an object (e.g. the central lid) to try to lever off the fixed lids. This behaviour is different from using an object to bang the box (which is coded under “tapping or banging”): the angle and pressure applied indicates that the child is trying to force *up* the top layer of the box. |
| *push side left* | Child pushes on fixed side of the box (left hand side) |
| *push side right* | Child pushes on fixed side of the box (right hand side) |
| **sitting / climbing on unit** | Child climbs or sits on unit |
| **crawling through unit** | Child crawls through table to which the box is fixed |
| **tapping or banging** | Child uses hands or another object/part of the box to bang/tap the box/unit |
| **licking** | Child licks the box (anywhere) |
| not touching* | Child’s fingers/hands/body are not touching box (accidental brushing of the box whilst moving past can be coded as not touching). |
| touching box but no obvious manipulation* | Child’s fingers/hands/body rest on the box (or attached table) but no pressure is applied and no other strategy is evident. |
| replacing lid* | Child replaces central compartment lid |
| replacing drawer* | Child pushes back drawer into position |
| replacing reward* | Child places reward back into the compartment after retrieval |
| re-retrieving reward* | Retrieving reward after it had already been retrieved at least once |
| strategy obscured* | Use this code only where strategy is entirely obscured. |

*these behaviours are included in overall task duration calculations but are not considered as goal-directed strategies, nor as sensory-motor exploration.

## Calculating Success Score

To calculate the Success Score the latency in seconds to retrieve each treat from task onset (the moment at which the researcher finished saying ‘Can you get the sweets’) was subtracted from 300 (the maximum task duration). A treat not being retrieved yielded a score of 0. The 3 scores were then summed to produce an overall Success Score such that a toddler who retrieved all 3 treats quickly achieved a score close to 900, and a toddler who retrieved no treats achieved a score of 0.

*Example 1: Child retrieves 1 reward, after 180 seconds, but no further reward*

Success Score = (300 – 180) + (300 – 300) + (300 – 300) = 120

*Example 2: Child retrieves 1 reward after 30 seconds, a second reward 50 seconds after the start of the task, and the third reward 120 seconds after the start of the task*

Success Score = (300 – 30) + (300 – 50) + (300 – 120) = 700.

Success Score was highly correlated with the number of rewards retrieved (*r_s_* =.915, *p* <.001) but captured greater variation by differentiating between children who retrieved the rewards quickly, and children who retrieved the rewards slowly.

## Coding reliability

Data were coded by 1 lead coder (MA) and 3 additional graduate student coders MH, BC, HC. STAARS and LonDownS data were coded by MA, MH and BC with an inter-rater reliability (assessed on a sub-set of 15 triple-coded videos) ranging from .746 (95% CI: .409 - .906) for Perseveration to .864 (CI: .653 - .952). TABLET data were coded by HC, who achieved an inter-rater reliability with MA (assessed on a sub-set of 7 (15%) (double-coded videos) ranging from .855, (95% CI: .374 – .973) for Persistence to .965 (95% CI: .811 – .994) for Perseveration. Study 2 data were coded by NK, who achieved an inter-rater reliability with MA (assessed on a sub-set of 35 double-coded videos) ranging from .902 (95% CI: .806 – .950) for Perseveration to .998 (95% CI: .996 – .999) for Success Score.

Supplementary Table 1.2 Correlations between task Success Score, Generativity, Persistence, and Perseveration: Study 1 (2- and 3-year-olds)

|  | Success Score | Generativity | Persistence |
| --- | --- | --- | --- |
| Generativity | .722 | - |  |
| Persistence | .503 | .641 | - |
| Perseveration | -.349 | -.442 | .138 |

*n* = 85-100, depending on variable; lowest for Perseveration as participants who engaged in less than 10 seconds of goal-directed manipulation were excluded for this variable. Pearson’s correlations used where both variables were normally-distributed (Generativity and Perseveration); Spearman’s rho used in all other instances.

Supplementary Table 1.3 Correlations between task Success Score, Generativity, Persistence, and Perseveration: Study 2 (4-year-olds)

|  | Success Score | Generativity | Persistence |
| --- | --- | --- | --- |
| Generativity | .646 | - |  |
| Persistence | .351 | .518 | - |
| Perseveration | -.116 | .001 | .745 |

*n* = 75-84, depending on variable; lowest for Perseveration as participants who engaged in less than 10 seconds of goal-directed manipulation were excluded for this variable. Spearman’s rho used for all correlations.

# Supplementary Materials 2: ECBQ and CBQ details, and associations with performance measures

In the CBQ and ECBQ IC scales parents are asked to report on how often they observed the child exhibiting the behaviours detailed in *Supplementary Table S2* during the last two weeks, on a scale of 1 (Never) to 7 (Always) . Items indicated with R are reverse coded.

*Supplementary Table 2.1* Items in the Inhibitory Control scales of the EBCQ and CBQ. Items shown in italics included in the full version of the ECBQ only.

| ECBQ | CBQ |
| --- | --- |
| *R. When asked NOT to, how often did your child touch an attractive item (such as an ornament) anyway?* | Approaches places s/he has been told are dangerous slowly and cautiously. |
| *R. When asked NOT to, how often did your child, run around your house or apartment anyway?* | Is good at following instructions. |
| R. When asked NOT to, how often did your child, play with something anyway? | R. Has trouble sitting still when s/he is told to (at movies, church, etc.). |
| When told “no”, how often did your child stop the forbidden activity? | Can easily stop an activity when s/he is told "no." |
| *When told “no”, how often did your child stop an activity quickly* | Prepares for trips and outings by planning things s/he will need. |
| *R. When told “no”, how often did your child ignore your warning* | Can wait before entering into new activities if s/he is asked to. |
| R. When asked to wait for a desirable item (such as ice cream), how often did your  child go after it anyway? |  |
| *When asked to wait for a desirable item (such as ice cream), how often did your child seem unable to wait for as long as 1 minute?* |  |
| When asked to wait for a desirable item (such as ice cream), wait patiently? |  |
| When asked to do so, how often was your child able to stop an ongoing activity? |  |
| When asked to do so, how often was your child able to lower his or her voice? |  |
| When asked to do so, how often was your child able to be careful with something breakable? |  |

Supplementary Table 2.1 Correlations between performance and parent-report measures of IC (Study 1)

|  | ECBQ IC | CBQ IC |
| --- | --- | --- |
| Glitter Wand: 2-year-olds (*n*=37) | .26 (.25)  [-.08, .56] | NA |
| Snack Delay: 3-year-olds (*n*=37) | NA | .11 (.14)  [-.24, .45] |

Cell values show uncorrected correlation co-efficient with 95% confidence interval in square parentheses, and correlation co-efficient controlling for Mullen visual reception normed score in round parentheses.

ECBQ: Early Childhood Behavior Questionnaire. CBQ: Children’s Behavior Questionnaire. IC: Inhibitory Control

# Supplementary Materials 3: Additional Analyses

## Age-related changes in components of problem-solving

To investigate the effect of development on the key variables associated with success on the task, we regressed Generativity, Persistence, Perseveration, and Success Score on age, in a series of linear regressions using a 1000-sample bootstrap procedure to accommodate the non-Gaussian distribution of the residuals for Success Score and Perseveration. To avoid potential confounds between age and nationality, these analyses were conducted within the Study 1 participants (UK cohorts from STAARS, LonDownS and TABLET projects) only.

As indicated in Supplementary Table 3.1 and Supplementary Figure S1, age was predictive of overall task performance (Success Score), whereby older children retrieved more rewards, faster, than younger children. Age was also predictive of Generativity, whereby older children produced more unique strategies than did younger children, and of Persistence, whereby older children spent a greater proportion of task time engaged in goal-directed manipulation than did younger children. A linear regression of age on Perseveration produced a poor model fit (see Supplementary Table 3.1), as did a quadratic model (*F*(2,81) = 1.48, *p* =.22, *R^2^* = .04).

Supplementary Table 3.1 Linear regressions of age on Success Score, Generativity, Persistence, and Perseveration

|  | *F* | *p* | *R^2^* | Standardised β | B [95% CI] |
| --- | --- | --- | --- | --- | --- |
| Success Score | *F*(1,97) = 13.64 | <.001 | .12 | .35 | .34 [156, .520] |
| Generativity | *F*(1,95) = 29.77 | <.001 | .24 | .49 | .01 [.004, .009] |
| Persistence | F(1,96) = 45.33 | <.001 | .32 | .57 | .00 [.000, .001] |
| Perseveration | *F*(1,82) = 1.43 | .24 | .02 | .13 | .00 [.000, .000] |

CI = Confidence Interval


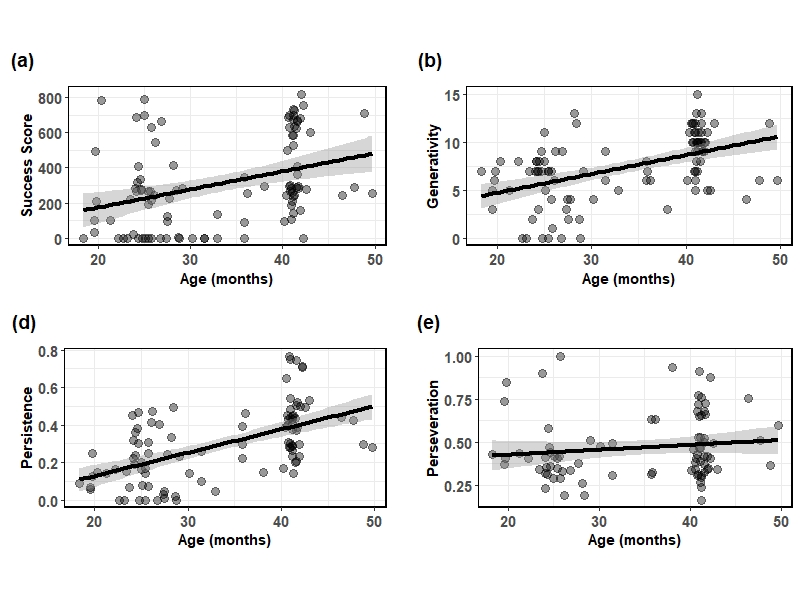


Supplementary Figure S1: Age-related changes in Problem-Solving Box task performance
